# Simultaneous boost radiotherapy versus conventional dose radiotherapy for patients with newly diagnosed glioblastoma: a multi-institutional analysis

**DOI:** 10.1038/s41598-024-60154-y

**Published:** 2024-04-23

**Authors:** Seiya Takano, Natsuo Tomita, Mayu Kuno, Masanari Niwa, Akira Torii, Taiki Takaoka, Nozomi Kita, Dai Okazaki, Shintaro Yamamoto, Tatsuya Kawai, Chikao Sugie, Yasutaka Ogawa, Kenichi Matsumoto, Kaoru Uchiyama, Shinya Otsuka, Tooru Matsui, Akifumi Miyakawa, Tomoki Mizuno, Masato Iida, Motoki Tanikawa, Mitsuhito Mase, Akio Hiwatashi

**Affiliations:** 1 Department of Radiology, Nagoya City University Graduate School of Medical Sciences, 1 Kawasumi, Mizuho-Cho, Mizuho-Ku, Nagoya, Aichi 467-8601 Japan; 2 Department of Radiation Oncology, Ichinomiya Municipal Hospital, 2-2-22 Bunkyo, Ichinomiya, Aichi 491-8558 Japan; 3 Department of Radiology, Japan Community Health Care Organization Chukyo Hospital, 1-1-10 Sanjo, Minami-Ku, Nagoya, Aichi 457-8510 Japan; 4 Department of Radiology, Nagoya City University Midori Municipal Hospital, 1-77 Shiomigaoka, Midori-Ku, Nagoya, Aichi 458-0037 Japan; 5 Department of Radiology, Japanese Red Cross Aichi Medical Center Nagoya Daini Hospital, 2-9 Myoken-Cho, Showa-Ku, Nagoya, Aichi 466-8650 Japan; 6 Department of Radiation Oncology, Kasugai Municipal Hospital, 1-1-1 Takaki-Cho, Kasugai, Aichi 486-8510 Japan; 7 Department of Radiation Oncology, Hokuto Hospital, 7-5 Kisen, Inada-Cho, Obihiro, Hokkaido, 080-0833 Japan; 8 Department of Radiology, Kariya Toyota General Hospital, 5-15 Sumiyoshi-Cho, Kariya, Aichi 448-8505 Japan; 9 Department of Radiology, Okazaki City Hospital, 3-1 Goshoai, Koryuji-Cho, Okazaki, Aichi 444-8553 Japan; 10 Department of Radiology, Konan Kosei Hospital, 137 Oomatsubara, Takaya-Cho, Konan, Aichi 483-8704 Japan; 11 Department of Radiation Oncology, National Hospital Organization Nagoya Medical Center, 4-1-1, Sannomaru, Naka-Ku, Nagoya, Aichi 460-0001 Japan; 12 Department of Radiation Oncology, Suzuka General Hospital, 1275-53 Yamanoue, Yasuzuka-Cho, Suzuka, Mie 513-0818 Japan; 13 Department of Radiology, Toyokawa City Hospital, Yawata-Cho Noji 23, Toyokawa, Aichi 442-8561 Japan; 14 Department of Neurosurgery, Nagoya City University Graduate School of Medical Sciences, 1 Kawasumi, Mizuho-Cho, Mizuho-Ku, Nagoya, Aichi 467-8601 Japan

**Keywords:** Radiotherapy, CNS cancer

## Abstract

We compared survival outcomes of high-dose concomitant boost radiotherapy (HDCBRT) and conventional dose radiotherapy (CRT) for newly diagnosed glioblastoma (GB). Patients treated with intensity-modulated radiation therapy for newly diagnosed GB were included. In HDCBRT, specific targets received 69, 60, and 51 Gy in 30 fractions, while 60 Gy in 30 fractions was administered with a standard radiotherapy method in CRT. Overall survival (OS) and progression-free survival (PFS) were compared using the Log-rank test, followed by multivariate Cox analysis. The inverse probability of treatment weighting (IPTW) method was also applied to each analysis. Among 102 eligible patients, 45 received HDCBRT and 57 received CRT. With a median follow-up of 16 months, the median survival times of OS and PFS were 21 and 9 months, respectively. No significant differences were observed in OS or PFS in the Kaplan–Meier analyses. In the multivariate analysis, HDCBRT correlated with improved OS (hazard ratio, 0.49; 95% confidence interval, 0.27–0.90; *P* = 0.021), and this result remained consistent after IPTW adjustments (*P* = 0.028). Conversely, dose suppression due to the proximity of normal tissues and IMRT field correlated with worse OS and PFS (*P* = 0.008 and 0.049, respectively). A prospective study with a stricter protocol is warranted to validate the efficacy of HDCBRT for GB.

## Introduction

The standard treatment for newly diagnosed glioblastoma (GB) involves surgery followed by radiation therapy (RT) and concurrent temozolomide (TMZ) in accordance with the Stupp protocol^1^. Randomized controlled trials conducted before the introduction of TMZ reported no additional clinical improvement beyond 60 Gy^2,3^. Based on these findings, a radiation dose of 60 Gy using conventional fractionation has been established as the current standard. However, there is still potential for higher local radiation doses to improve outcomes in GB patients because the predominant pattern of treatment failure remains local even in the era of TMZ^4,5^.

Since 2012, our institutions have implemented high-dose concomitant boost radiotherapy (HDCBRT) as a treatment option. HDCBRT delivers a locally escalated radiation dose of 69 Gy in 30 fractions to the surgical cavity and residual tumors. Previous studies demonstrated the dosimetric benefits of simultaneous integrated boost intensity-modulated radiation therapy (SIB-IMRT) for delivering high doses while sparing normal tissues^6,7^. Improved survival outcomes with local dose escalations using SIB have recently been reported^8,9^. However, a limited number of studies have directly compared survival outcomes between local dose escalations using SIB and the standard RT dose regimen following the Stupp protocol. Therefore, the aim of this multi-institutional study was to compare the survival outcomes of patients with newly diagnosed GB who received HDCBRT and conventional dose RT (CRT).

## Methods

### Patient population

The medical records of patients treated with IMRT for newly diagnosed GB between January 2012 and December 2022 at 11 institutions were reviewed. A total of 106 patients were initially screened for eligibility based on the following inclusion criteria: (1) histologically confirmed GB (the 2007/2016 World Health Organization [WHO] grade IV or 2021 WHO grade 4), (2) the concurrent use of TMZ, (3) the use of IMRT in all fractions, and (4) the delivery of at least 90% of the planned total dose. Patients who received IMRT with a fraction dose deviating by 0.2 Gy or more from the dose specified in each protocol (*n* = 3) and those who did not receive boost irradiation (*n* = 1) were subsequently excluded because of the difficulties associated with classifying and comparing these cases between both groups. This yielded a final cohort of 102 eligible patients; 45 received HDCBRT and 57 received CRT.

The present study was conducted in accordance with the Declaration of Helsinki and its later amendments. This study was approved by each institutional review board of Nagoya City University Graduate School of Medical Sciences (3/13/2023, No. 60–22-0132), Ichinomiya Municipal Hospital (7/20/2023, No. 1356), Japan Community Health care Organization Chukyo Hospital (4/4/2023, No. 2022060), Japanese Red Cross Aichi Medical Center Nagoya Daini Hospital (5/19/2023, No. 1585), Kasugai Municipal Hospital (5/23/2023, No 23–6), Hokuto Hospital (6/14/2023, No. 2023–1121-R1), Kariya Toyota General Hospital (4/13/2023, No. 859), Okazaki City Hospital (4/7/2023, No. 2023–02), Konan Kosei Hospital (5/11/2023, No. 2022–052), National Hospital Organization Nagoya Medical Center (4/21/2023, No. 2023–002), and Toyokawa City Hospital (4/26/2023, No. 186). Since this was a retrospective study, the Nagoya City University Ethics Committee waived the need for informed consent as part of the study approval in line with the Ethical Guidelines for Medical and Health Research Involving Human Subjects in Japan. The research content was disclosed in the form of opt-out on the website.

### Treatment planning and target delineation

Treatment planning was performed using a non-contrast CT fused with T2-weighted image or fluid-attenuated inversion recovery as well as contrast-enhanced T1-weighted image from a postoperative magnetic resonance (MR) imaging. IMRT was initiated using 6- to 10-MV photon beams with multiple static intensity-modulated beams (Clinac 2100 CD, Varian Medical Systems, Palo Alto, CA, USA; Clinac iX, Varian Medical Systems, Palo Alto, CA, USA), intensity-modulated dynamic arcs (Trilogy, Varian Medical Systems, Palo Alto, CA, USA; TrueBeam, Varian Medical Systems, Palo Alto, CA, USA), or helical tomotherapy (TomoTherapy, Accuray Inc., Sunnyvale, CA, USA; Radixact, Accuray Inc., Sunnyvale, CA, USA). Patients were immobilized in a supine position using a thermoplastic mask.

**Figure 1:**
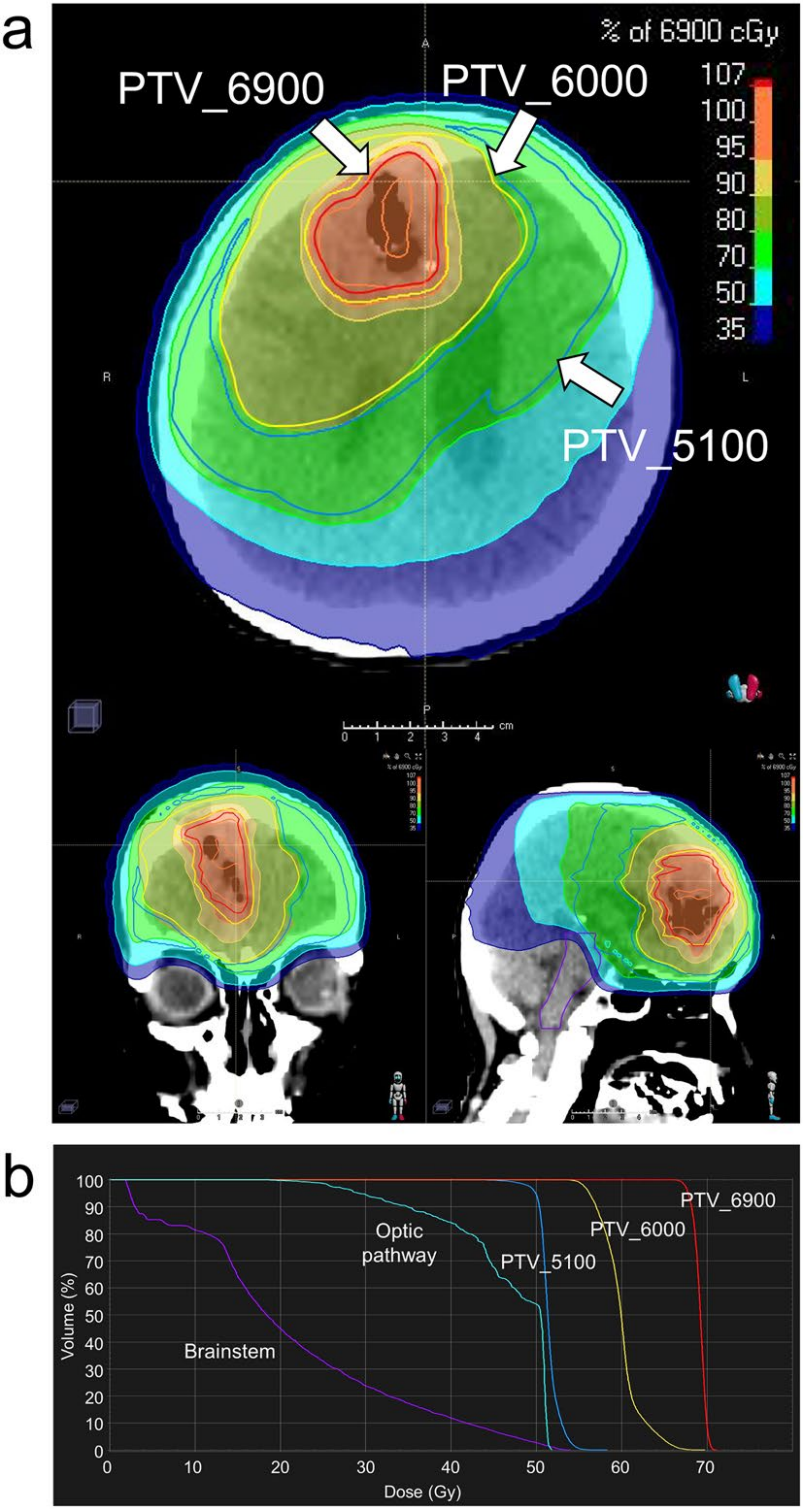
(**a**) A representative dose distribution of high-dose concomitant boost radiotherapy (HDCBRT). Planning target volume_6900 (PTV_6900) outlined in red encompasses contrast-enhancing residual tumors and the surgical cavity. PTV_6900 is identical to the gross tumor volume (GTV). PTV_6000 outlined in yellow, includes the GTV with a 1.5-cm margin and surrounding edema, both of which are further expanded with a 2- to 3-mm margin. PTV_5100 outlined in blue, includes the GTV with a 4-cm margin and surrounding edema with a 2-cm margin, both of which are further expanded with a 2- to 3-mm margin. (**b**) A dose-volume histogram of HDCBRT. PTV_6900, PTV_6000, and PTV_5100 received 69, 60, and 51 Gy in daily fractions of 2.3, 2.0, and 1.7 Gy, respectively.

In HDCBRT, the gross tumor volume (GTV) was defined as contrast-enhancing residual tumors and the surgical cavity. The clinical target volume (CTV) was not used. Planning target volume_6900 (PTV_6900) was identical to the GTV. PTV_6000 included the GTV with a 1.5-cm margin and surrounding edema, both of which were further expanded with a 2- to 3-mm margin. PTV_5100 included the GTV with a 4-cm margin and surrounding edema with a 2-cm margin, both of which were further expanded with a 2- to 3-mm margin. Surrounding edema was identified on T2-weighted sequences. Total doses of 69, 60, and 51 Gy were prescribed to PTV_6900, PTV_6000, and PTV_5100 using the D50% prescription in daily fractions of 2.3, 2.0, and 1.7 Gy, respectively. PTVs were manually trimmed based on anatomical boundaries. Dose constraints were as follows: Dmax < 50 Gy for the optic nerves and chiasm (alternatively, Dmax < 54 Gy if not achievable); Dmax < 54 Gy for the brainstem (alternatively, Dmax < 64 Gy, D33% < 60 Gy, and D66% < 53 Gy if not achievable); Dmax < 40 Gy for the eyes; and Dmax < 10 Gy for the lenses. A dose suppression to 51 Gy was allowed within the PTV that overlapped with the optic pathway and/or brainstem. Figure 1 shows a representative dose distribution of HDCBRT.

In CRT, target volumes were generally delineated according to the recent Japan Clinical Oncology Group trial^10^. GTV was defined as contrast-enhancing residual tumors. CTV_initial included the GTV, surgical cavity, and surrounding edema identified on T2-weighted images with a 1.5- to 2.0-cm margin. CTV_boost included the GTV and surgical cavity with a 1.5- to 2.0-cm margin along with surrounding edema. PTV_initial and PTV_boost were defined as CTV_initial and CTV_boost with a 5-mm margin, respectively. In three out of 54 patients, target volume delineation followed the Radiation Therapy Oncology Group (RTOG) guidelines. Initial and boost irradiation were performed using two-step (40–50 Gy to PTV_initial and 60 Gy to PTV_boost in 2-Gy fractions) or SIB techniques (45–54 Gy in 30 daily fractions to PTV_initial and 60 Gy in 30 daily fractions to PTV_boost) using the D50% prescription, except for one patient who received two-step irradiation with the mean dose prescription. Dose constraints for normal tissues were at the discretion of each radiation oncologist.

### Surgery and chemotherapy

IMRT was initiated without delay following surgery or biopsy. The extent of resection was stratified into gross total resection, subtotal resection, or biopsy only. Gross total resection was defined as the complete removal of contrast-enhancing tumors on a postoperative MR images. At the time of resection, Gliadel wafers (Eisai Co. Ltd., Tokyo, Japan) were placed in the resection cavity at the discretion of each neurosurgeon. In principle, all patients received concurrent TMZ (75 mg/m^2^/day) during the course of RT. Adjuvant TMZ was administered for 5 consecutive days every 28 days after the completion of RT (150 mg/m^2^/day during the first cycle and 200 mg/m^2^/day during subsequent cycles)^1^. The duration of adjuvant TMZ was selected at the discretion of each neurosurgeon.

### Outcomes

The primary endpoint was overall survival (OS), calculated from the date of surgery until death from any cause. Progression-free survival (PFS) was calculated from the date of surgery until either tumor progression or death. Tumor progression was defined based on a modified version of the Macdonald criteria^11^: new enhancing lesions in the intracranial space, a ≥ 25% increase in the sum of the products of the perpendicular diameters of contrast-enhancing lesions, or the initiation of secondary treatment for clinically evident local relapse. Local failure was defined as progression within PTV_boost, PTV_6900, or PTV_6000, regional failure as progression within PTV_initial or PTV_5100, distant failure as dissemination or progression outside the PTV, and multifocal progression as the occurrence of both local or regional failure and distant failure. The follow-up duration was calculated from the date of surgery. Radiation necrosis following IMRT was assessed according to Common Terminology Criteria for Adverse Events version 5.0. MR perfusion and MR spectroscopy were not routinely conducted unless tumor progression or radiation necrosis was suspected.

### Statistical analysis

Patient and treatment characteristics were compared using Fisher’s exact test or the chi-square test for categorical variables and the Mann–Whitney U test for continuous variables. The Kaplan–Meier method was used to calculate OS and PFS rates, and survival estimates were compared using the Log-rank test between the HDCBRT and CRT groups. Cox proportional hazards models were used for univariate and multivariate analyses. The initial sites of failure were compared using Fisher’s exact test. In univariate and multivariate analyses, survival estimations, and the Log-rank test, the inverse probability of treatment weighting (IPTW) method, utilizing propensity scores, was employed to balance the observed differences in baseline characteristics between the two treatment groups. The propensity score was defined as the probability of a patient receiving HDCBRT and was calculated from a multivariate logistic regression model including the following covariates: Age, sex, the tumor location, Karnofsky performance status, extent of resection, postoperative neurological function, the isocitrate dehydrogenase 1 (IDH1) mutation status, O^6^-methylguanine-DNA methyltransferase (MGMT) status, 1p19q codeletion status, MIB-1 index, time from surgery to RT, and year of surgery. The weight of the IPTW adjustment for each patient was defined as Z/*e* + (1‒Z)/*e*, where Z is a binary variable (Z = 1 for HDCBRT and Z = 0 for CRT) and *e* is a propensity score^12^. Recursive partitioning analysis classification was performed according to a previous study^13^. All statistical analyses were conducted using R (version 3.6.3; R Foundation for Statistical Computing, Vienna, Austria). The threshold for significance was *P* < 0.05.

## Results

### Patient characteristics

Table 1 shows patient characteristics before applying IPTW. The percentages of the MIB-1 index, IDH1 mutation, and MGMT methylation statuses significantly differed between the HDCBRT and CRT groups (*P* = 0.001, 0.010, and 0.031, respectively). Dose suppression within the PTV-organs at risk (OAR) overlap was significantly more common in patients receiving HDCBRT than in those receiving CRT (73% vs. 30%, *P* < 0.001, Table 2). PTV volumes were significantly higher in the HDCBRT group (*P* < 0.008). There was no significant difference in the rate of Gliadel wafer implantation between the two groups (*P* = 0.10). The rate and duration of adjuvant TMZ were similar between the two groups. After applying IPTW, there was an overall decrease in standardized differences in patient characteristics (Table 3). No significant differences were observed in patient or treatment characteristics, except for the boost technique, total doses, and the presence of dose suppression within the PTV-OAR overlap (Tables 3 and 4).

**Table 1:** Patient characteristics before applying the inverse probability of treatment weighting.

Characteristics		All (*n* = 102)	HDCBRT (*n* = 45)	CRT (*n* = 57)	*P*-value	Standardized difference
Sex	Male/Female	64 (63%)/38 (37%)	24 (53%)/21 (47%)	40 (70%)/17 (30%)	0.10	0.35
Age (years)	Median (range)	68 (7–83)	68 (24–79)	68 (7–83)	0.52	0.039
Tumor location					0.58	0.44
	Frontal/temporal	38 (37%)/28 (28%)	18 (40%)/13 (29%)	20 (35%)/15 (26%)		
	Parietal/occipital/cerebellum	19 (19%)/5 (5%)/6 (6%)	5 (11%)/2 (4%)/4 (9%)	14 (25%)/3 (5%)/2 (4%)		
	Multifocal/others	3 (3%)/3 (3%)	2 (4%)/1 (2%)	1 (2%)/2 (4%)		
KPS	90–100/70–80/ ≤ 60	33 (32%)/37 (36%)/32 (31%)	17 (38%)/13 (29%)/15 (33%)	16 (28%)/24 (42%)/17 (30%)	0.37	0.29
Extent of resection	GTR/STR/Biopsy	44 (43%)/45 (44%)/13 (13%)	21 (47%)/20 (44%)/4 (9%)	23 (40%)/25 (44%)/9 (16%)	0.58	0.22
Postoperative neurological function	Able to work/not	41 (40%)/61 (60%)	18 (40%)/27 (60%)	23 (40%)/34 (60%)	1.0	0.007
RPA classification	III/IV/V	33 (32%)/1 (1%)/68 (67%)	17 (38%)/0 (0%)/28 (62%)	16 (28%)/1 (2%)/40 (70%)	0.45	0.27
MIB-1 index	≤ 30%/ > 30%/unknown	43 (42%)/38 (37%)/21 (21%)	24 (53%)/19 (42%)/2 (4%)	19 (33%)/19 (33%)/19 (33%)	0.001	0.80
IDH1 mutation	Yes/No/unknown	7 (7%)/49 (48%)/46 (45%)	0 (0%)/19 (42%)/26 (58%)	7 (12%)/30 (53%)/20 (35%)	0.010	0.65
MGMT methylation	Yes/No/unknown	2 (2%)/9 (9%)/91 (89%)	0 (0%)/1 (2%)/44 (98%)	2 (4%)/8 (14%)/47 (83%)	0.031	0.54
1p19q codeletion	Yes/No/unknown	0 (0%)/22 (22%)/80 (78%)	0 (0%)/6 (13%)/39 (87%)	0 (0%/16 (28%)/41 (72%)	0.092	0.37
Time from surgery to radiation therapy (days)	< 20/ ≥ 20	48 (47%)/54 (53%)	16 (36%)/29 (64%)	32 (56%)/25 (44%)	0.047	0.42
Year of surgery	2012–2017/2018–2022	41 (40%)/61 (60%)	19 (42%)/26 (58%)	22 (39%)/35 (61%)	0.84	0.074

HDCBRT, high-dose concomitant boost radiotherapy; CRT, conventional dose radiotherapy; KPS, Karnofsky performance status; GTR, gross total resection; STR, subtotal resection; RPA, recursive partitioning analysis; IDH1, isocitrate dehydrogenase 1; MGMT, O^6^-methylguanine-DNA methyltransferase.

Data are n (%) or medians (range).

**Table 2:** Treatment characteristics before applying the inverse probability of treatment weighting.

Characteristics		All (*n* = 102)	HDCBRT (*n* = 45)	CRT (*n* = 57)	*P*-value
Boost technique	SIB/two-step	70 (69%)/32 (31%)	45 (100%)/0 (0%)	25 (44%)/32 (56%)	< 0.001
GTV volume (mL)	Median (range)	37 (0–360)	37 (0–270)	39 (0–360)	0.98
PTV volume (mL)	Median (range)	585 (106–1142)	656 (189–1007)	549 (106–1142)	0.008
Total dose (Gy)	59.2/60/69	1 (1%)/56 (55%)/45 (44%)	0 (0%)/0 (0%)/45 (100%)	1 (2%)/56 (98%)/0 (0%)	< 0.001
Fraction number (fractions)	30/29	101 (99%)/1 (1%)	45 (100%)/0 (0%)	56 (98%)/1 (2%)	1.0
Dose suppression within the PTV-OAR overlap		50 (49%)	33 (73%)	17 (30%)	< 0.001
Maximum dose (Gy)	Median (range)	51.0 (37.4–60.0)	51.0 (37.4–60.0)	51.0 (47.6–54.0)	0.74
OAR	BS/OP*/BS + OP	25 (50%)/4 (8%)/21 (42%)	16 (49%)/3 (9%)/14 (42%)	9 (53%)/1 (6%)/7 (41%)	1.0
Adjuvant TMZ	Yes/No/unknown	84 (82%)/17 (17%)/1 (1%)	38 (84%)/7 (16%)/0 (0%)	46 (81%)/10 (18%)/1 (2%)	0.80
Adjuvant TMZ (cycles)	Median (range)	6 (0–80)	6 (0–41)	5 (0–80)	0.28
Bevacizumab		29 (28%)	14 (31%)	15 (26%)	0.66
Gliadel wafer		36 (35%)	20 (44%)	16 (28%)	0.10
Tumor treating fields		2 (2%)	2 (4%)	0 (0%)	0.19
Follow-up duration (months)	Median (range)	16 (1– 93)	18 (2– 69)	13 (1–93)	0.11

HDCBRT, high-dose concomitant boost radiotherapy; CRT, conventional dose radiotherapy; SIB, simultaneous integrated boost; GTV, gross tumor volume; PTV, planning target volume; OAR, organs at risk; BS, brainstem; OP, optic pathway; TMZ, temozolomide.

Data are n (%) or medians (range).

*The optic pathway includes the optic nerves and chiasm.

**Table 3:** Patient characteristics after applying the inverse probability of treatment weighting.

Characteristics		All (n = 92.4)	HDCBRT (n = 39.0)	CRT (n = 53.4)	*P*-value	Standardized difference
Sex	Male/female	62.7 (68%)/29.6 (32%)	26.0 (67%)/12.9 (33%)	36.7 (69%)/16.7 (31%)	0.86	0.042
Age (years)	Median (range)	68 (7–83)	67 (24–79)	68 (7–83)	0.38	0.035
Tumor location					1.0	0.14
	Frontal/temporal	40.4 (44%)/17.1 (19%)	16.0 (41%)/7.1 (18%)	24.3 (46%)/10.0 (19%)		
	Parietal/occipital/cerebellum	18.4 (20%)/3.9 (4%)/6.2 (7%)	8.3 (21%)/1.4 (4%)/3.2 (8%)	10.1 (19%)/2.5 (5%)/3.1 (6%)		
	Multifocal/others	3.1 (3%)/3.3 (4%)	1.4 (4%)/1.5 (4%)	1.6 (3%)/1.8 (3%)		
KPS	90–100/70–80/ ≤ 60	23.9 (26%)/36.6 (40%)/31.9 (35%)	10.7 (28%)/14.5 (37%)/13.8 (35%)	13.1 (25%)/22.1 (41%)/18.2 (34%)	0.93	0.093
Extent of resection	GTR/STR/Biopsy	31.4 (34%)/44.0 (48%)/16.9 (18%)	12.6 (33%)/17.6 (45%)/8.7 (22%)	18.8 (35%)/26.4 (50%)/8.2 (15%)	0.77	0.18
Postoperative neurological function	Able to work/not	29.3 (32%)/63.0 (68%)	11.8 (30%)/27.2 (70%)	17.6 (33%)/35.8 (67%)	0.79	0.058
RPA class	III/IV/V	23.9 (26%)/0.9 (1%)/67.6 (73%)	10.7 (28%)/ 0.0 (0%)/28.2 (73%)	13.1 (25%)/ 0.9 (2%)/39.4 (74%)	0.69	0.19
MIB-1 index	≤ 30%/ > 30%/Unknown	40.6 (44%)/34.0 (37%)/17.7 (19%)	17.9 (46%)/15.1 (39%)/6.0 (15%)	22.8 (43%)/18.9 (35%)/11.7 (22%)	0.83	0.17
IDH1 mutation	Yes/No/unknown	3.9 (4%)/ 44.6 (48%)/ 43.8 (48%)	0.0 (0%)/18.3 (47%)/20.7 (53%)	3.9 (7%)/26.3 (49%)/23.2 (43%)	0.22	0.42
MGMT methylation	Yes/No/unknown	1.1 (1%)/8.0 (9%)/83.2 (90%)	0.0 (0%)/ 2.8 (7%)/36.2 (93%)	1.1 (2%)/5.2 (10%)/47.1 (88%)	0.62	0.23
1p19q codeletion	Yes/No/unknown	0.0 (0%)/17.1 (19%)/75.2 (81%)	0.0 (0%)/ 5.6 (14%)/33.4 (86%)	0.0 (0%)/11.5 (22%)/41.9 (78%)	0.39	0.19
Time from surgery to radiation therapy (days)	< 20/ > = 20	42.9 (46%)/49.5 (54%)	18.5 (48%)/20.4 (53%)	24.4 (46%)/29.0 (54%)	0.88	0.038
Year of surgery	2012–2017/2018–2022	30.8 (33%)/61.6 (67%)	12.7 (33%)/26.3 (67%)	18.1 (34%)/35.3 (66%)	0.91	0.028

HDCBRT, high-dose concomitant boost radiotherapy; CRT, conventional dose radiotherapy; KPS, Karnofsky performance status; GTR, gross total resection; STR, subtotal resection; RPA, recursive partitioning analysis; IDH1, isocitrate dehydrogenase 1; MGMT, O6-methylguanine-DNA methyltransferase.

Data are n (%) or medians (range).

**Table 4:** Treatment characteristics after applying the inverse probability of treatment weighting.

Characteristics		All (*n* = 92.4)	HDCBRT (*n* = 39.0)	CRT (*n* = 53.4)	*P*-value
Boost technique	SIB/two-step	61.7 (67%)/30.7 (33%)	39.0 (100%)/0 (0%)	22.7 (43%)/30.7 (58%)	< 0.001
GTV volume (mL)	Median (range)	34 (0–360)	34 (0–270)	36 (0–360)	0.81
PTV volume (mL)	Median (range)	581 (106–1142)	579 (189–1007)	586 (106–1142)	0.50
Total dose (Gy)	59.2/60/69	0.8 (1%)/52.6 (57%)/39.0 (42%)	0 (0%)/0 (0%)/39.0 (100%)	0.8 (2%)/52.6 (99%)/0 (0%)	< 0.001
Fraction number (fractions)	30/29	91.5 (99%)/0.8 (1%)	39.0 (100%)/0 (0%)	52.6 (99%)/0.8 (2%)	0.40
Dose suppression within the PTV-OAR overlap	44.6 (48%)	25.1 (65%)	19.5% (37%)	0.047	
Maximum dose (Gy)	Median (range)	51.0 (37.4–60.0)	51.0 (37.4–60.0)	52.6 (47.6–54.0)	0.97
OAR	BS/OP */BS + OP	21.3 (48%)/2.6 (6%)/20.7 (46%)	12.5 (50%)/2.0 (8%)/10.5 (42%)	8.8 (45%)/0.6 (3%)/10.1 (52%)	0.66
Adjuvant TMZ	Yes/No/unknown	72.2 (79%)/19.6 (21%)/0.5 (0%)	31.6 (81%)/7.4 (19%)/0 (0%)	40.7 (77%)/12.2 (23%)/0.5 (0%)	0.71
Adjuvant TMZ (cycles)	Median (range)	6 (0–80)	7 (0–41)	5 (0–80)	0.095
Bevacizumab		32 (35%)	14.9 (38%)	17 (32%)	0.61
Gliadel wafer		25.7 (28%)	12.7 (33%)	13.0 (24%)	0.40
Tumor treating fields		1.8 (2%)	1.8 (5%)	0 (0%)	0.11
Follow-up duration (months)	Median (range)	16 (1–93)	18 (2–69)	13 (1–93)	0.09

HDCBRT, high-dose concomitant boost radiotherapy; CRT, conventional dose radiotherapy; SIB, simultaneous integrated boost; GTV, gross tumor volume; PTV, planning target volume; OAR, organs at risk; BS, brainstem; OP, optic pathway; TMZ, temozolomide.

Data are n (%) or medians (range).

*The optic pathway includes the optic nerves and chiasm.

### Outcomes

**Figure 2:**
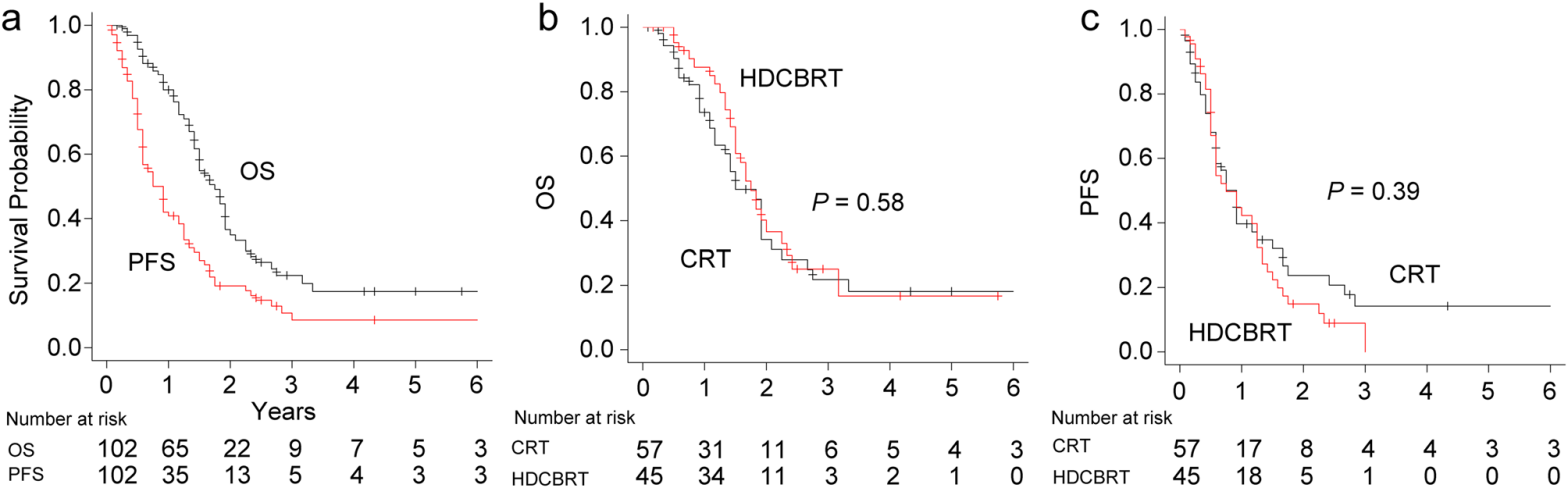
(**a**) Survival curves of overall survival (OS) and progression-free survival (PFS) in all patients. Comparisons of OS (**b**) and PFS (**c**) between the high-dose concomitant boost radiotherapy (HDCBRT) and conventional dose radiotherapy (CRT) groups before applying the inverse probability of treatment weighting.

The median follow-up time was 16 months (range 1–93 months) for all patients and 13 months (range 1–93 months) for living patients. By the last follow-up visit, 59 (58%) patients had died and 78 (76%) showed disease progression. Figure 2 shows the survival curves of OS and PFS before IPTW adjustments. The median survival times of OS and PFS in all patients were 21 months (95% confidence interval [CI], 18–23) and 9 months (95% CI 7–14), respectively (Fig. 2a). The median survival time of OS was 21 months (95% CI 18–27) in HDCBRT and 18 months (95% CI 14–23) in CRT, with no significant differences (*P* = 0.58, Fig. 2b). One- and 2-year OS rates were 88 and 37% in HDCBRT and 74 and 34% in CRT, respectively. The median survival time of PFS was 9 months (95% CI 7–15) in HDCBRT and 11 months (95% CI 7–15) in CRT, with no significant differences (*P* = 0.39, Fig. 2c). One- and 2-year PFS rates were 42 and 15% in HDCBRT, and 40 and 24% in CRT, respectively. After IPTW adjustments, no significant differences in OS or PFS survival curves were observed between the HDCBRT and CRT groups (*P* = 0.36 for OS and *P* = 0.98 for PFS; see Supplementary Figure S1).

The results of univariate and multivariate analyses predicting OS after IMRT are shown in Table 5. The multivariate analysis showed the independent effects of dose suppression within the PTV-OAR overlap (hazard ratio [HR], 2.93; 95% CI 1.54–5.56; *P* = 0.001), age ≥ 68 years (HR 2.35; 95% CI 1.34–4.16; *P* = 0.003), and HDCBRT (HR 0.49; 95% CI 0.27–0.90; *P* = 0.021) on OS before IPTW adjustments. In the IPTW-adjusted multivariate analysis, HDCBRT still correlated with improved OS (HR 0.50; 95% CI 0.27–0.93; *P* = 0.028). In the IPTW-adjusted multivariate analysis, dose suppression within the PTV-OAR overlap was the only significant prognostic factor for progression (HR 1.68; 95% CI 1.00–2.80; *P* = 0.049; Table 5).

**Table 5:** Univariate and multivariate analyses of OS and PFS after IMRT.

Predictor	OS				PFS			
	Univariate		Multivariate		Univariate		Multivariate	
	HR (95% CI)	*P-*value	HR (95% CI)	*P-*value	HR (95% CI)	*P-*value	HR (95% CI)	*P-*value
Before IPTW adjustments								
Age ≥ 68 years	1.63 (0.98–2.74)	0.061	2.35 (1.34–4.16)	0.003	1.06 (0.67–1.66)	0.81	1.15 (0.72–1.83)	0.56
Dose suppression within the PTV-OAR overlap	1.65 (0.99–2.77)	0.057	2.93 (1.54–5.56)	0.001	1.53 (0.97–2.40)	0.067	1.58 (0.95–2.63)	0.081
KPS ≤ 80	0.96 (0.56–1.65)	0.89	0.65 (0.36–1.18)	0.16	0.96 (0.60–1.54)	0.87	0.83 (0.50–1.37)	0.46
STR/biopsy vs. GTR	1.23 (0.73–2.06)	0.45	1.21 (0.71–2.07)	0.49	1.25 (0.80–1.98)	0.33	1.31 (0.81–2.11)	0.27
Gliadel wafer	1.09 (0.64–1.87)	0.75	1.24 (0.71–2.17)	0.44	1.08 (0.68–1.72)	0.76	1.12 (0.70–1.81)	0.63
HDCBRT vs. CRT	0.87 (0.52–1.45)	0.75	0.49 (0.27–0.90)	0.021	1.21 (0.77–1.89)	0.41	0.99 (0.60–1.65)	0.98
After IPTW adjustments								
Age ≥ 68 years	1.35 (0.79–2.30)	0.27	1.89 (1.06–3.38)	0.032	0.99 (0.62–1.57)	0.96	1.13 (0.70–1.83)	0.61
Dose suppression within the PTV-OAR overlap	1.64 (0.96–2.79)	0.071	2.30 (1.24–4.28)	0.008	1.59 (0.98–2.57)	0.058	1.68 (1.00–2.80)	0.049
KPS ≤ 80	0.97 (0.53–1.78)	0.93	0.71 (0.37–1.35)	0.30	0.92 (0.54–1.56)	0.76	0.79 (0.46–1.36)	0.40
STR/biopsy vs. GTR	1.39 (0.79–2.45)	0.26	1.56 (0.86–2.83)	0.14	1.39 (0.84–2.29)	0.20	1.50 (0.87–2.56)	0.14
Gliadel wafer	1.26 (0.70–2.28)	0.44	1.40 (0.77–2.57)	0.27	1.13 (0.67–1.91)	0.63	1.18 (0.70–2.00)	0.54
HDCBRT vs. CRT	0.75 (0.43–1.29)	0.30	0.50 (0.27–0.93)	0.028	0.99 (0.62–1.60)	0.98	0.81 (0.49–1.34)	0.41

OS, overall survival; PFS, progression-free survival; IMRT, intensity-modulated radiation therapy; HR, hazard ratio; CI, confidence interval; IPTW, inverse probability of treatment weighting; PTV, planning target volume; OAR, organs at risk; KPS, Karnofsky performance status; STR, subtotal resection; GTR, gross total resection; HDCBRT, high-dose concomitant boost radiotherapy; CRT, conventional dose radiotherapy.

In the HDCBRT group, two patients (4%) developed grade ≥ 2 radiation necrosis. One patient exhibited grade 2 radiation necrosis, which was confirmed through a pathological diagnosis following reoperation due to the progression of contrast-enhancing lesions on MR imaging. The other patient developed grade 3 radiation necrosis, diagnosed based on contrast-enhanced MR imaging. In both cases, radiation necrosis was observed within PTV_6900. No patients in the CRT group developed grade ≥ 2 radiation necrosis.

### Patterns of failure

Among the 78 patients with disease progression, one patient showed the progression of intracranial lesions, which was clinically estimated by the attending physician. Thirteen patients had died before evident tumor progression, while radiological imaging identified tumor progression in 64 patients. The initial sites of failure were as follows: local failure in 45 patients (70%), regional failure in 7 (11%), distant failure in 9 (14%), and multifocal failure in 3 (5%). No significant differences were observed in the distribution of initial failure sites between the HDCBRT and CRT groups (*P* = 0.20, see Supplementary Table S1).

## Discussion

In the present multi-institutional study, we compared survival outcomes between HDCBRT and CRT. No significant differences were observed in OS or PFS in the Kaplan–Meier analyses between the two groups (Figs. 2 and S1). However, the multivariate analysis showed that HDCBRT correlated with better OS (HR 0.49; 95% CI 0.27–0.90; *P* = 0.021; Table 5) after adjusting for the impact of dose suppression within the PTV-OAR overlap. By applying IPTW, we aimed to minimize any potential selection bias in the allocation of patients to HDCBRT or CRT. The benefit of HDCBRT on OS was still significant in the IPTW-adjusted multivariate analysis (HR 0.50; 95% CI 0.27–0.93; *P* = 0.028; Table 5).

Conflicting findings have been reported on the survival outcomes of local dose escalations using SIB-IMRT^8,9,14,15^. In a recent retrospective study, moderate dose escalations using SIB-IMRT of up to 66 Gy in 30 fractions achieved superior OS and intracranial control to the standard dose regimen^9^. However, in a phase II randomized controlled trial comparing a hypofractionated accelerated SIB-IMRT arm (60 Gy in 20 fractions) with a three-dimensional conformal radiotherapy arm (60 Gy in 30 fractions), no significant difference was observed in survival outcomes although there was a trend toward better survival outcomes in the hypofractionated arm^14,16^. Possible factors contributing to these differences include variations in the biologically effective dose. Although GB cells are radioresistant, radiobiological analyses revealed a correlation between the total dose and tumor control^17,18^. One example is a large retrospective study, in which dose escalations up to 80–90 Gy significantly prolonged OS, but resulted in an unacceptably high incidence of brain necrosis^19^. The total dose used in the present study corresponded to 71.7 Gy in an equivalent dose at 2 Gy per fraction (EQD2) with an α/β ratio of 5.6^20^, and was higher than those previously utilized in similar studies (range 63.2–67.9 Gy in EQD2)^9,14,15,20^. Although this high dose may have contributed to the improvement in OS, the extent of dose escalations may still have been insufficient to control tumor progression because the pattern of recurrence was predominantly local (Supplementary Table S1). In contrast to the findings of a similar retrospective study^9^, we did not observe significant differences in PFS between the two treatment groups (Table 5). One possible explanation is that the definition of tumor progression used in the present study may have included early post-irradiation pseudo-progression^11^. Additionally, in the present study, a routine evaluation using contrast-enhanced MRI was not performed. This may have led to the underreporting of tumor progression events.

As a secondary result, dose suppression within the PTV-OAR overlap emerged as an independent prognostic factor for worse OS and PFS (Table 5). To the best of our knowledge, the negative impact of dose suppression within the PTV on clinical outcomes has not yet been reported, although total doses < 60 Gy have been shown to correlate with decreased OS^21,22^. According to the NRG consensus guidelines, the brainstem and optic pathway within 2 cm of T1 contrast enhancement need to receive a dose of approximately 54 Gy, with a planning organ at risk volume Dmax < 55 Gy^23^. In the present study, the maximum achieved dose constraints for the optic pathway and brainstem were slightly lower (median of 51 Gy) than those of the NRG guidelines, which may have been due to the lack of a specific goal for PTV dose coverage. Unexpectedly, dose suppression within the PTV-OAR overlap was more frequent in patients receiving HDCBRT than in those receiving CRT (Table 2, 73% vs. 30%, *P* < 0.001). Therefore, a lower radiation dose within the PTV may have masked the effect of local dose escalations on OS in the Kaplan–Meier analyses (Figs. 2 and S1). Higher prescribed doses and a lower proportion of tumors located in the parietal and occipital regions (15% for HDCBRT and 30% for CRT, Table 1), which are usually located away from critical organs, may have led to frequent dose suppression in HDCBRT. Another possible reason is that the HDCBRT protocol explicitly allowed a dose suppression to 51 Gy within the PTV overlapping with the optic pathway and/or brainstem, which may have influenced the physician’s decision to reduce doses. A prospective study with specific rules for PTV coverage and OAR dose constraints is warranted.

Differences in target delineation between the HDCBRT and CRT groups also need to be considered. PTV_5100 in HDCBRT was partly identical to PTV_initial in CRT in target delineation because the prescribed dose to PTV_5100 was 49 Gy in EQD2 (α/β = 5.6), which is comparable to that to PTV_initial. The PTV was expanded beyond surrounding edema, based on the principle that recurrence predominantly occurs within a 2.0-cm margin of the primary site^24^. The PTV volume in HDCBRT was slightly larger than that in CRT, probably because PTV_5100 always included the GTV with a 4-cm margin in addition to edema with a 2-cm margin. However, a randomized clinical trial showed that different target volume delineations according to the RTOG and European Organization for Research and Treatment of Cancer (EORTC) did not significantly affect clinical outcomes or patterns of failure despite the use of a single and smaller PTV in the EORTC protocol^4,25^. A retrospective study also revealed no significant differences in failure patterns between 5, 10, and 15–20 mm CTV margins^26^. Therefore, the differences in target delineation and size may not have a substantial impact on survival outcomes in the present study.

Our findings suggested that local dose escalation using SIB may improve survival outcomes (Table 5). Recent studies have suggested promising approaches using new imaging modalities, such as ^18^F-fluoroethyltyrosine positron emission tomography (^18^F-FET PET) and 3,4-dihydroxy-6-[18F]-fluoro-L-phenylalanine PET (^18^F-DOPA PET), to achieve more effective local dose escalation^27,28^. These techniques combined with SIB have the potential to deliver higher doses to biologically active tumor cells with a reduced CTV margin^27^. However, the incidence of radiation necrosis remains high despite the use of these techniques (13% for grade 3 radiation necrosis)^28^. Similarly, in the present study, grade ≥ 2 radiation necrosis occurred only in the HDCBRT group and was localized in PTV_6900 in all patients (*n* = 2). This finding is also partially consistent with a retrospective study showing that grade ≥ 2 necrosis occurred only in patients receiving a total dose of ≥ 72 Gy in 30 fractions^29^. Therefore, further investigation is warranted to optimize dose fractionation to minimize adverse effects, in addition to more sophisticated target volume delineation.

The present study has several limitations. Due to its retrospective design, the results obtained were affected by unmeasured confounders despite the application of IPTW. Unmeasured confounders included comorbidities such as cardiac and cerebrovascular disease, which affect short-term mortality. We also did not evaluate the use of corticosteroids for cerebral edema. Corticosteroids have been reported to decrease OS through protective effects against treatment-related genotoxic stress^30^. Tumors located in the deep nuclei or basal ganglia can also be confounders associated with worse survival outcomes^31^. Additionally, in principle, we were unable to adjust for covariates identified after treatment allocation, such as treatment characteristics. However, treatment characteristics between the two groups were nearly homogenous, except for the use of dose suppression within the PTV-OAR overlap after IPTW adjustments. Furthermore, the lack of a specific protocol defining PTV coverage, OAR sparing, and optimization parameters, along with the lack of availability of detailed DVH data, made it difficult to discuss the individual impacts of the SIB technique and local dose escalations on clinical outcomes. Therefore, prospective dose-escalation studies using SIB would require stringent protocols to ensure PTV coverage and manage adjacent critical OARs. Moreover, specific molecular subtyping, including the assessment of MGMT promoter methylation and IDH1 mutation status, was not routinely performed in this study due to the lack of insurance coverage in our country. Therefore, we were unable to assess the prognostic significance of the MGMT promotor methylation or IDH1 mutation statuses^32,33^.

In conclusion, in the present study, the multivariate analysis demonstrated that HDCBRT was independently associated with improved OS in patients with newly diagnosed GB after adjusting for the negative impact of dose suppression within the PTV-OAR overlap on both OS and PFS. The benefit of HDCBRT for OS remained consistent in the IPTW-adjusted multivariate analysis. A prospective study with a stricter protocol is warranted to validate the efficacy of HDCBRT as a treatment for GB.

### Supplementary Information

Supplementary Information.

## Data Availability

The datasets generated and/or analyzed during the present study cannot be accessed publicly for ethical reasons, but may be obtained from the corresponding author upon reasonable request.
