# Supplementary material for: Simultaneous boost radiotherapy versus conventional dose radiotherapy for patients with newly diagnosed glioblastoma: a multi-institutional analysis
Source: Sci Rep. 2024 Apr 23;14:9283. doi: 10.1038/s41598-024-60154-y (PMC11039761; doi:10.1038/s41598-024-60154-y)
Supplement: Supplementary file 1 — Supplementary Information. [file 41598_2024_60154_MOESM1_ESM.docx]

**Supplementary Table S1** Initial sites of failure

|  | **HDCBRT (n = 32)** | **CRT (n = 32)** | ***P*-value** |
| --- | --- | --- | --- |
| **Local** | 20 (63%) | 25 (78%) | 0.20 |
| **Regional** | 6 (19%) | 1 (3%) |  |
| **Distant** | 5 (16%) | 4 (13%) |  |
| **Multifocal** | 1 (4%) | 2 (6%) |  |

HDCBRT, high-dose concomitant boost radiotherapy; CRT, conventional dose radiotherapy.

Local failure was defined as progression within planning target volume_boost (PTV_boost), PTV_6900, or PTV_6000. Regional failure was defined as progression within PTV_initial or PTV_5100. Distant failure was defined as either dissemination or progression occurring outside the PTV. Multifocal progression was defined as the occurrence of both local or regional failure and distant failure.


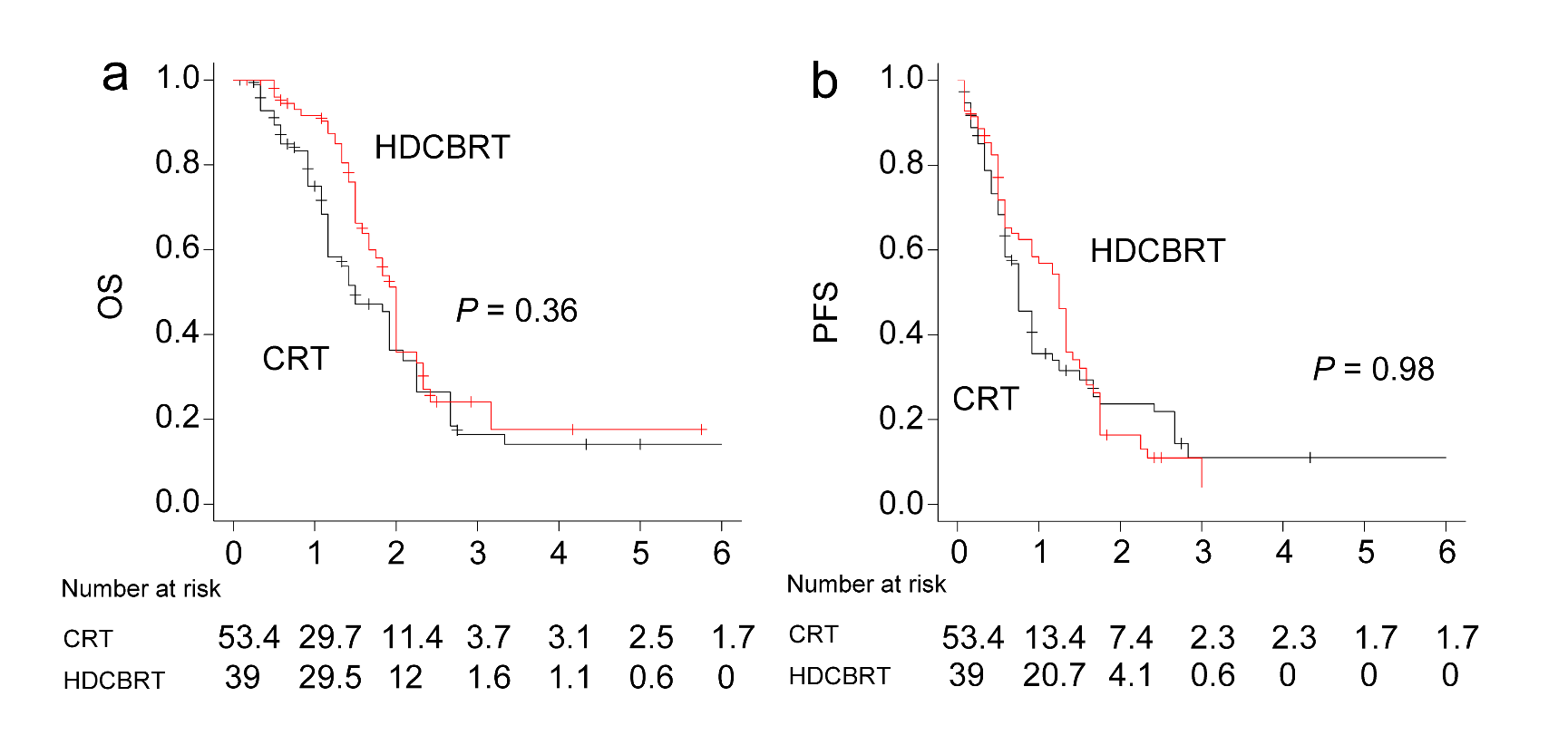


**Supplementary Figure S1** Comparisons of overall survival (OS) (a) and progression-free survival (PFS) (b) between the high-dose concomitant boost radiotherapy (HDCBRT) and conventional dose radiotherapy (CRT) groups after applying the inverse probability of treatment weighting.
